# What enables and hinders nursing staff in delivering person-centred fundamental care? A qualitative study within the incharge programme

**DOI:** 10.1186/s12912-025-04038-0

**Published:** 2025-10-31

**Authors:** Anna-Karin Gunnarsson, Therese Avallin, Anna Hauffman, Lena Nyholm, Katarina Edfeldt, Camilla Fröjd, Elin Björk, Eva Jangland

**Affiliations:** Nursing Research, Department of Surgical Sciences, Uppsala University, Uppsala University Hospital, Uppsala, S-751 85 Sweden

**Keywords:** Person-centred care, Nurse, Qualitative, Leadership, Fundamental care, Culture, Routines

## Abstract

**Background:**

Meeting patients’ fundamental care needs is a core aspect of nursing. However, there is compelling evidence of missed nursing care within surgical care. Person-centred care is known to increase patient involvement and prevent missed nursing care. The Fundamentals of Care framework aligns with person-centred care principles. It has the essential relationship-based care process at its core and serves as a guide for delivering person-centred fundamental care. This study aims to describe what enables and hinders the delivery of person-centred fundamental care as identified by registered nurses and nursing assistants in a surgical department.

**Methods:**

A qualitative descriptive study was performed. Registered nurses and nurse assistants (nursing staff) from three surgical wards at a university hospital participated in a workshop package about person-centred fundamental care. Data were collected from two workshops with 106 and 92 participants, respectively, individual written reflections on a situation in clinical practice related to person-centred fundamental care delivery (83 participants), and three focus group interviews with a total of 13 participants. The data were analysed using content analysis.

**Results:**

The results present the nursing staffs’ descriptions of what enables and hinders the delivery of person-centred fundamental care in three categories: [1] a culture of daily mentorship to ensure adherence to person-centred routines; [2] a distinct nursing leadership—supportive in both routines of daily practice and high-pressure situations; and [3] enhancing comprehension of person-centred care routines to embed the approach in everyday thinking and actions.

**Conclusions:**

To provide person-centred fundamental care, nursing staff need organizational support in both culture and routines. Nurse managers need to be clear on how care is to be delivered, challenge prevailing old habits, and act as role models in changing practices. Nursing staff need to take their responsibility by adhering to routines and contributing to driving changes. The results can be used to navigate the complex implementation of person-centred fundamental care and also demonstrate the need for a change toward person-centred fundamental care being valued and prioritized in organizations.

**Clinical trial number:**

Not applicable.

**Supplementary Information:**

The online version contains supplementary material available at 10.1186/s12912-025-04038-0.

## Background

Fundamental care needs are the universal needs shared by all individuals, regardless of their health status or illness [1–3]. Meeting a patient’s fundamental care needs—such as nutrition, mobilization, and dignity—is critical not only for respecting the patient but also for ensuring safety and preventing adverse outcomes, including infections and delayed wound healing. Unmet needs can lead to increased suffering, prolonged hospital stay, and increased healthcare costs [4–7]. Surgical care is evolving rapidly thanks to advanced surgical techniques, enabling care and curing of patients with advanced surgical illnesses. Furthermore, advanced surgeries are being performed in more fragile and older patients, and hospital stays are becoming shorter, increasing the demands on nursing care [8, 9]. Nursing care that meets each patient’s fundamental care needs is essential to enable the patient to be safe, involved, and informed [10–14]. However, there is compelling evidence of missed nursing care within surgical care, including that patients do not have their fundamental care needs met and are not being involved in their care to ensure that it meets their needs and preferences [15–17]. Failure to approach nursing as a holistic care process and instead reducing it to a series of separate tasks can be part of the reason that tasks are overlooked [18]. Focusing on each task separately leaves little space for the performance of person-centred care using the essential relationship-based care process.

Person-centred care is known to be crucial for patient care needs to be met. The body of knowledge on person-centred care is extensive, and researchers have strived to define person-centred care. A recent report [19] provides an overview of current knowledge in the field, including several theoretical frameworks. Although person-centred care has evolved over the past two decades, the terms *person-centred care* and *patient-centred care* are still often used interchangeably [20]. The review by Håkansson et al. (2019) distinguishes between the two, noting that patient-centred care emphasizes supporting patients living a functional life, whereas person-centred care focuses on patients living a meaningful life. Central to person-centred care is the principle of mutual respect and collaboration between healthcare professionals and patients, where individual care needs are acknowledged, and the patients are actively involved as partners in their care [19].

Despite the benefits of a person-centred approach to care, healthcare organizations struggle in their quest to implement person-centred care [21, 22], and studies across multiple countries report missed nursing care [10, 23, 24]. The Fundamentals of Care framework aligns with person-centred care principles. It has the essential relationship-based care process at its core and serves as a guide for delivering person-centred fundamental care. The conceptual framework is intended to guide the holistic care process with practical recommendations for delivering person-centred fundamental care [1–3]. Literature on application of the framework in different settings and countries is growing [25]. The framework was developed in response to global concerns regarding unmet fundamental care needs of patients, to guide clinicians, managers, and policy stakeholders to meet those needs [26]. The international development is grounded in seminal work, including a narrative review of nursing literature, patient interviews, and a Delphi study [3, 26, 27]. Feo et al. (2018) define fundamental care as: ‘*Actions on the part of the nurse that respect and focus on a person’s essential needs to ensure their physical and psychosocial wellbeing. These needs are met by developing a positive and trusting relationship with the person being cared for as well as their family/carers*’ (citation Feo et al. 2018, Table 3, page 2295). The framework comprises three interconnected dimensions: [1] a trusting relationship between care recipient and care provider [2], integrating and meeting the care recipient’s physical and psychosocial care needs, and the care provider’s relational actions in recognizing and managing those needs, and [3] a context of care that is supportive of relationship development and care integration [1, 3]. An illustration of the framework is available in a number of languages on the International Learning Collaborative’s website [28]. The Swedish version of the framework was used in the present study [29] and has also been published in a textbook on how to apply the framework in clinical practice [30].

The dimension of contextual factors in the Fundamentals of Care framework includes factors at the system and policy level that are needed to deliver holistic and safe care [1, 3, 14]. The dimension encompasses multiple care delivery elements, such as resources (e.g., routines, staff, competence utilization), leadership (informal and formal), culture (e.g., values and norms within an organization), and regulations [3]. The contextual factors act at three levels: (micro (individual factors), meso (ward/department factors), and macro (broad policy-level factors) [31]. Thus, the factors interact with stakeholders at both the individual level, such as nursing staff, and the organizational level, such as nurse managers. Contextual factors can be used to understand and uncover the reasons behind the outcome of fundamental care delivery within an organization [3].

Healthcare should deliver person-centred fundamental care to patients. Extensive work needs to be performed within organizations to transform care. Meeting patients’ fundamental care needs is the professional responsibility of registered nurses and is a core aspect of nursing, requiring nurses to understand and utilize the person-centred fundamental care process [3].There is a need for a change toward person-centred fundamental care within surgical care; however, this transformation has been found to be challenging, in part due to high patient turnover and registered nurses’ heavy workload [32]. To achieve a change, we need to understand what enables and hinders nursing staff from delivering person-centred fundamental care. In this study, we sought the perspectives of nursing staff, including registered nurses (RNs) and nurse assistants (NAs), on the enablers and obstacles to providing person-centred fundamental care to patients cared for in surgical wards.

## Methods

### Aim

This study aimed to describe what enables and hinders the delivery of person-centred fundamental care as identified by RNs and NAs in a surgical department.

### Design

**Fig. 1:**
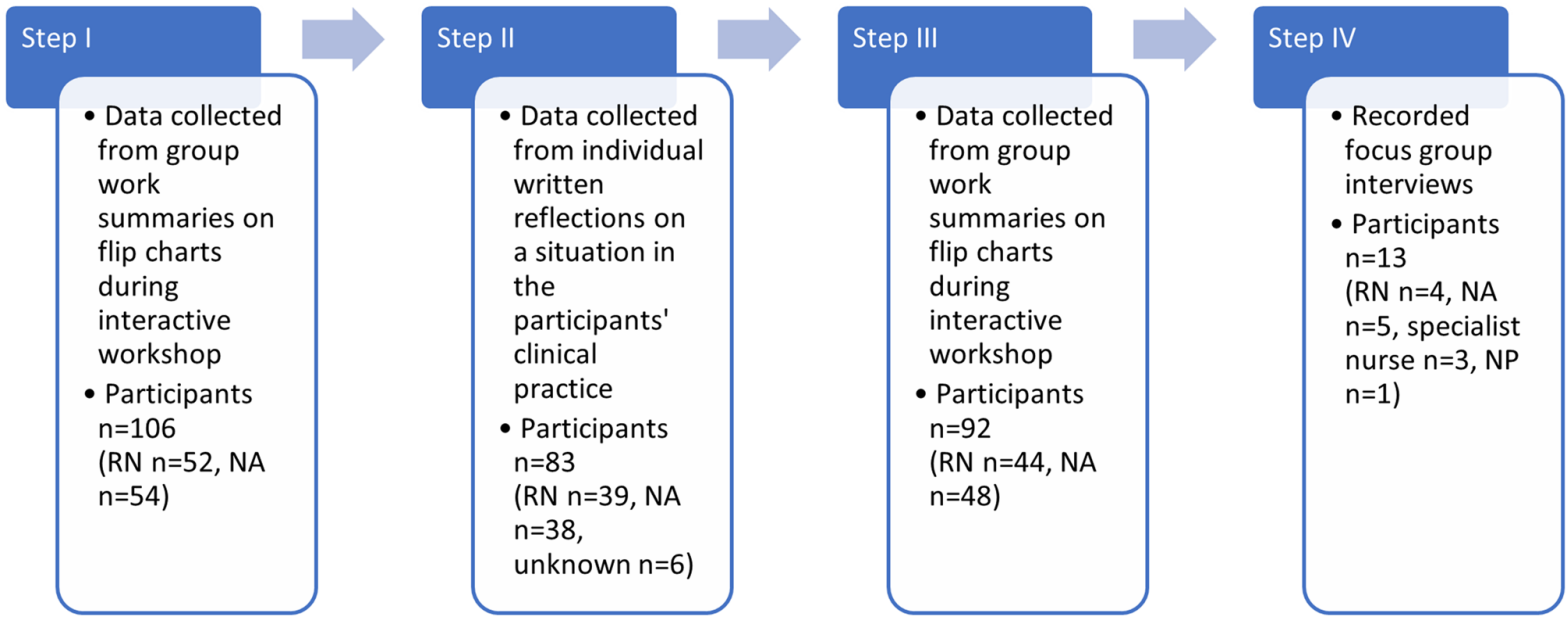
The four steps of data collection and the participants included. RN = registered nurse, NA = nurse assistant, NP = nurse practitioner

A qualitative descriptive study using a four-step procedure to collect data, see Fig. 1. The descriptive design was preferred because it stays close to and captures participants’ experiences and yields findings reflecting those experiences [33]. The study is part of the action research programme InCHARGE (Innovations to utilize nurses’ competence and achieve person-centred care—Fundamentals of Care goes into practice), theoretically grounded in the Fundamentals of Care framework [3, 34]. The Consolidated Criteria for Reporting Qualitative Research (COREQ) [35] were used to ensure sufficient reporting.

### Study setting

The participants were employed at three wards in a surgical department at a Swedish university hospital, where adult patients from the region, nation, or other countries were cared for following acute or planned admission for abdominal, vascular, or endocrine surgical illness. Lectures on the Fundamentals of Care Framework had been delivered over the course of several years; however, no strategic education initiative had been undertaken involving all three wards. Over the past decade, routines based on the Fundamentals of Care framework had been implemented on the wards. All wards had implemented nursing rounds performed by the clinical nurse specialist, and one ward also had a person-centred team round and a bedside shift handover, aligned with the framework. However, compliance with these routines was inconsistent.

Advanced surgery was performed at the department, which included two centres of excellence. Each patient was cared for by an RN (with a three-year bachelor’s degree) and an NA (with a three-year education in upper secondary school). At the time of the data collection, each RN and NA pair was jointly responsible for 5–7 patients during day and evening shifts and 12 patients during night shifts. The surgeon conducted daily rounds, visited patients postoperatively and at discharge meetings, and was available for on-call duties. Specialist nurses (with a one-year master’s degree) worked on all three wards and across all shifts, although there was not always a specialist nurse on duty during every single shift. Nurse practitioners (with a two-year master’s degree) were available on two wards, Monday through Friday, from 8 am to 4 pm. At the time of the study, agency RNs were hired to work on two wards. On each ward, a nurse manager (RN, specialist nurse) and two or three assistant nurse managers (RNs, specialist nurses) were responsible for nursing care and staffing. Academic nursing leadership, including a Head of Nursing (RN, specialist nurse, PhD) and a Head of Nursing Research (RN, specialist nurse, associate professor), was established in the department when the study was initiated. Each ward had beds for 24 patients; however, owing to the nursing shortage, up to 12 beds were closed, with the number varying daily.

An evidence-based workshop package on person-centred fundamental care, guided by the Fundamentals of Care framework, was performed in the department during the fall of 2022. The workshop package was part of an action research cycle [35]. The workshops aimed to educate and engage the nursing staff and initiate thought processes among them regarding person-centred fundamental care and person-centredness in connection with their professional practices. The workshops mentioned contextual factors such as roles, responsibilities, and collaboration in the care team and values underpinning nursing care. The workshop package included two interactive workshops, where short lectures were interspersed with interactive group discussions, based on the assumption that active work increases learning [36]. The lectures and interactive group discussions focused on the Fundamentals of Care framework, person-centred care, and the person-centred care approach [1–3, 16, 30, 37, 38]. The presentation also included previous results from the surgical department regarding missed nursing care and oral care [10, 39], and the patient perspective. Additionally, individual reflections on a situation in clinical practice regarding person-centred fundamental care delivery were included. The workshops were co-created by the research team and clinical specialist nurses representing the surgical department, some of whom had knowledge about the framework. Three researchers (AKG, TA, and EJ) from the research team, all with extended knowledge about the framework, served as moderators during the interactive workshops at the hospital.

### Participants

All RNs (including specialist nurses and nurse practitioners) and NAs permanently employed at the wards and attending the workshops were, at the start of the workshops, invited to participate in the study through verbal and written information, provided face-to-face by the moderators. A total of 112 out of 134 workers (employed in spring 2022) participated. The intention was for all participants to attend both workshops; however, due to illness, vacation, and unplanned clinical work, this did not happen. It is unknown how many participants attended both workshops.

For the focus group interviews, convenience samples were used, which included 13 participants. One interview included 3 participants (RN *n* = 3) and two interviews included 5 participants each (RN *n* = 2, NA *n* = 3; RN *n* = 3, NA *n* = 2). The exclusion criterion was not attending any workshop. The demographic data for the participants were as follows: RNs (*n* = 8), number of years in their profession: mean 8.5 years, range 0.5–21 years; and years on the ward: mean 7.4 years; range 0.5–21 years. NAs (*n* = 5), number of years in their profession: mean 16, range 1.5–20; and years on the ward: mean 8.7, range 0.5–20. To preserve participant anonymity, specialist nurses and nurse practitioners have not been identified in the demographic data, as such specification could potentially allow individuals familiar with the wards to infer their identities.

### Data collection

Data were collected through flip charts during interactive workshops, through individual written reflections on a situation in the participants’ clinical practice, and through recordings of focus group interviews (Fig. 1). The workshops were conducted in an auditorium located within the hospital, whereas the focus group interviews took place in conference rooms located within each respective ward. The questions and interview guide were developed within this study (Supplementary file). The questions discussed in Step I were developed based on the aim of the study and in collaboration with the clinical specialist nurses representing the surgical department. In Step II, the questions were designed to allow participants to apply the knowledge gained in Step I. The questions in Step III were inspired by the discussions shared by the participants during Step I. The questions for the focus group interviews were formulated after Step III and were grounded in the findings from Steps I and III. On the flip charts, the participants summarized their interactive group discussions during the workshops with single words or short sentences. Each group consisted of participants from the same ward. During Step I (September to October 2022), 23 flip charts were collected (words *n* = 3,053). During Step III (November to December 2022), another 23 flip charts were collected, encompassing 845 words. A structured interview guide with open-ended questions was used during the workshops (Supplementary file). During Step I, the questions dealt with what person-centred routines existed on the ward, the enablers of and hindrances for the routines, and the nursing staff’s own roles in these routines. The questions also focused on what routines they wanted to continue working with and their own roles in achieving that goal. In the second workshop, Step III, the questions dealt with what the nursing staff wanted their ward to develop further and what was required to succeed.

In the individuals’ written reflections on a situation in their clinical practice, Step II (November to December 2022), the participants were given 15 min to describe a situation they had personally chosen to observe and their reflections related thereto. A structured guide with open-ended questions was used (Supplementary file). The questions to reflect on were what in the situation was or was not person-centred, what enabled and hindered person-centredness in the situation, and what their role was in making the situation person-centred. A total of 83 written reflections were collected, each ½–1 page long. These reflections provided participants with an opportunity to apply the knowledge they had gained during Workshop I in the context of their clinical practice.

Three focus group interviews (Step IV) were recorded (February 2023), one for each ward. The interview guide for the focus groups was adapted to each respective ward, with the content being based on the results from Steps I and III for that ward (Supplementary file). The guide focused on what enabled and hindered nursing staff’s delivery of person-centred fundamental care in five areas, as emerged in Steps I and III: the person-centred approach; person-centred routines, prerequisites to using a person-centred approach at work, roles in the ward to strengthen fundamental care, and relevance and feasibility. During the interviews, the participants had all the questions in front of them, and they chose what parts to discuss within each question area. Two representatives from the research team conducted each focus group interview (AKG, TA, and EJ); one moderated the discussion, and one took field notes and asked additional questions. The focus group interviews lasted for 72–88 min. The interviews were audio-recorded, and demographic data were collected in writing (age, years in occupation, years at the surgical clinic, and highest level of education).

### Data analysis

All data were analysed using manifest content analysis to stay close to the text and maintain a low degree of interpretation [40]. After completion of the workshops in Steps I and III, the collected data (single words or short sentences) were transcribed verbatim by AKG and TA, and analysed by AKG, TA, and EJ. Five question areas were identified and subsequently used in the focus group interviews conducted in Step IV (see Interview guide in Supplementary file).

**Fig. 2:**
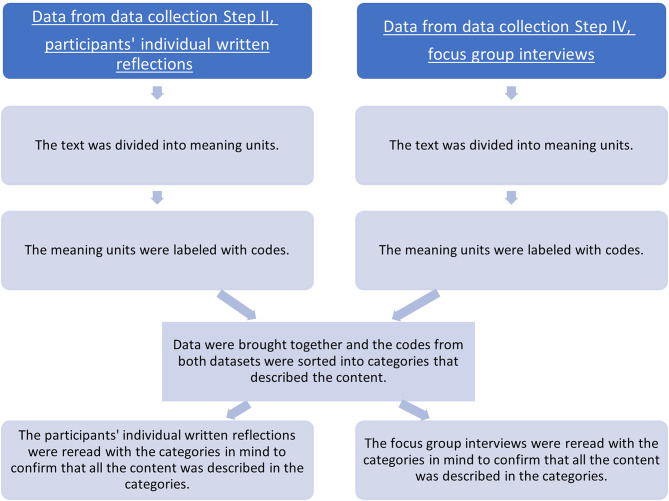
Process of the data analysis of data from participants’ individual written reflections, Step II, and the focus group interviews, Step IV

**Fig. 3:**
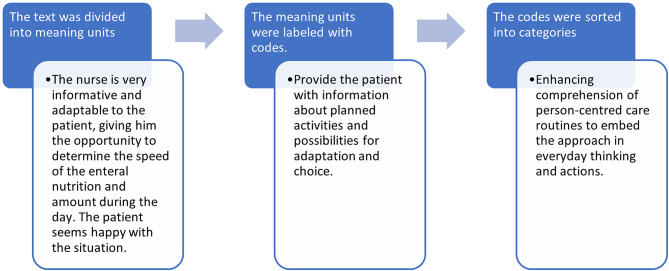
Example of the data analysis of a participant’s individual written reflection (Step II)

Once all three focus group interviews had been conducted, the data from Steps II and IV were analysed. Data from Step II were transcribed verbatim by AKG and TA, and data from Step IV by a professional transcription service. To initiate the analysis, the text was read several times to gain a sense of the whole. Field notes were also read. Step II was analysed by TA and Step IV by AKG and EJ separately. Both steps were then analysed jointly by AKG, EJ and TA (Fig. 2). The results were discussed several times within the research group (AKG, EJ, TA, AH, LN, KE, CF) so a consensus could be reached. Microsoft Excel was used for data organization and analysis. An example of the data analysis is presented in Fig. 3. The results from the focus group interviews and participants’ individual written reflections were not reported back to the participants for validation. Information regarding the expertise of the researchers responsible for the data analysis is presented in the supplementary file.

### Ethical considerations

The Swedish Ethical Review Authority requires no ethical approval for studies involving care providers when no physical procedures are involved, if there is no risk of physical or psychological harm, or if no sensitive personal data (e.g., political opinions or religious beliefs) are collected [41]. In this study, no sensitive data were collected. The study was conducted in accordance with the ethical principles outlined in the Declaration of Helsinki [42]. All participants signed a consent form. The participants were informed verbally and in writing that their participation in the study was voluntary and that they could withdraw from the study at any time by contacting one of the researchers without explanation or consequences (no participant chose to withdraw). The head of the surgical department approved the study. The workshop package was part of the wards’ in-job training; therefore, great care was taken to ensure that the nursing staff were informed about voluntary participation. Also, participation in the workshop was possible without participating in the study; a number of workshop participants declined to participate in the study. For example, it was possible to participate less actively in discussions and to omit performing the individual written reflection.

## Results

The results present the nursing staffs’ descriptions of what enabled and hindered the delivery of person-centred fundamental care in three categories: [1] a culture of daily mentorship to ensure adherence to person-centred routines; [2] a distinct nursing leadership—supportive in both routines of daily practice and high-pressure situations; and [3] enhancing comprehension of person-centred care routines to embed the approach in everyday thinking and actions. A summary of what enabled and hindered the delivery of person-centred fundamental care, as identified by nursing staff, is presented in Table 1. The result is illustrated with quotes from the nursing staff’s individual written reflections following their clinical observations and from the focus group interviews.

**Table 1:** Overview of categories and a summary of what enables and hinders the delivery of person-centred fundamental care. Examples are presented in the main text

A culture of daily mentorship to ensure adherence to person-centred routines	A distinct nursing leadership—supportive in both routines of daily practice and high-pressure situations	Enhancing comprehension of person-centred care routines to embed the approach in everyday thinking and actions
***What enables is…*** …an open culture in the ward, with an inclusive, collaborative learning environment and teamwork …a person-centred approach embedded in the culture of the ward …a continuous need for reminders to adhere to the routines	***What enables is…*** …visible nursing leadership from the nurse managers …a strengthened leadership role for the RNs in the care team …clear definitions of responsibilities in different roles	***What enables is…*** …increased knowledge and understanding of the difference between person-centred care and ‘traditional’ care …the nursing staff’s own leadership and motivation to acquire sufficient knowledge
***What hinders is…***	***What hinders is…***	***What hinders is…***
…lack of time and/or resources, vague routines, and colleagues who do not follow the routines …a task-oriented culture …when fundamental care needs are considered secondary to medical care needs	… when nurse managers themselves fill staffing gaps due to sick leave	…nursing staff who find it difficult when patients are expected to be involved in person-centred routines …when there is an attitude of futility and not all team members have the same understanding of person-centred care

### A culture of daily mentorship to ensure adherence to person-centred routines

The participants acknowledged person-centred fundamental care as relevant and essential to their daily practice, and they presented both enablers and hindrances to such care. The participants shared their current or previous person-centred routines on the wards, and routines enabling person-centred fundamental care across the wards were identified. These routines, such as the person-centred team round at the bedside, were described as beneficial for both patients and staff, as all care team members, including the patient, got access to the same information simultaneously, and the patient was involved in care delivery. Lack of time and/or resources, vague routines, and colleagues who did not follow the routines—or were not inclined to change their routines or approaches—were identified as hindering the successful implementation of person-centred care. One participant said that:

*It is a challenge we face: the patient needs to undergo monitoring and receive breakfast*,* and in the midst of all this*,* the ward rounds take place.* (NA, focus group interview)

Rounds took place in the middle of the NAs’ bedside care, and some participants shared that they sometimes did not prioritize attending them for this reason. Participants said that the surgeon and the RN might have found it easier to conduct the rounds in the nurses’ station and inform the patients of their decisions afterward.

The participants expressed a need for an improved, more open culture in the ward, where everyone felt comfortable asking questions, supporting each other, and readily giving and receiving constructive feedback and guidance. One example was when the team report (bedside shift handover) took too long because a colleague did not follow the routine. However, instead of providing constructive feedback to that colleague and allowing them to try again, nothing was said. The participants shared that this incident served as a convenient excuse for others to discontinue adherence to the routine, and the routine was allowed to lapse altogether.

The nursing staff stated that to enable person-centred fundamental care, it is essential that the patient is active in the care team when identifying care needs and how to meet those needs. It was emphasized that patients should be informed about their treatment and care, know what is happening to them, feel safe and respected, and feel trust for nursing staff. Several routines that already supported person-centred care were described, e.g., the team round at the bedside. However, the participants also described a lack of adherence to these routines, resulting in person-centred care not being available to all patients. A hindrance was identified in a written reflection where the patient’s fundamental care needs were described as being considered secondary to medical care needs. This prioritization of medical needs negatively affected care quality in several cases; for example, fundamental care needs were overlooked during rounds, leading to more extended hospital stays or patients being discharged before their fundamental care needs, such as toileting, were met. In a written reflection following a clinical observation, it was described that the decision to discharge a patient was made on medical grounds, without talking to the patient, who had loose stools during their morning bowel movement. Repeatedly, reflections stressed the importance of visibly integrating the fundamental care needs into the patient’s care plan and prioritizing those needs, from patient admission until discharge. The participants mentioned that the person-centred approach was essential for enabling person-centred routines but that they only ‘*had that approach partly*’ in the wards. One NA stated that:

*One must adopt the approach in order to fully grasp the importance of doing things in alternative ways.* (NA, focus group interview)

For example, during ward rounds, the importance of drinking nutritional supplements might be mentioned briefly, but the patient should receive further information in a follow-up discussion. However, such follow-up discussions were often skipped. In all focus group interviews, a task-oriented culture was described as hindering a person-centred approach and routines. The participants stated that they wanted to apply a person-centred approach in their work by involving the patient in the dialogue or setting shared goals for the day. However, the participants described a lack of support from routines and culture as hindering that ambition. The task focus, lack of understanding of the person-centred approach and routines, lack of time, stress of the job, and lack of cooperation from colleagues were identified as hindering aspects. One participant described the task-focused approach, attributing it to stress:

*I think it’s difficult to consider the patient as part of the team*,* as a human being. You only really see the patients*,* or some of them at least*,* as a task and that this task has to be completed and then you leave. It’s like*,* I think you forget about it*,* especially when there’s a high stress factor. At least*,* that’s what I think.* (RN, focus group interview)

Team communication was shown to enable the delivery of person-centred fundamental care on busy wards. Routines such as the team report and the team rounds were described as helping to ensure that all team members, including the patient, shared the same information at the same time without anything being lost or delayed. The teamwork was presented as enabling person-centred care. However, it was noted that sticking to routines was not easy, and there was a continuous need for reminders to adhere to the routines chosen. An RN expressed this as follows:

*I think having the patient as part of the team is great. Because that’s what it’s really all about. When the other teamwork [nurse/assistant nurse] isn’t working properly*,* it’s kind of hard to include the patient in an already disorganized team.* (RN, focus group interview)

### A distinct nursing leadership—supportive in both routines of daily practice and high-pressure situations

The participants asked for distinct and adaptable operative leadership in daily practice from the nurse managers, as a prerequisite to enabling, implementing, and sustaining person-centred routines. The participants described a need to strengthen the professional role of the RNs so the nurses could use their competence to enable person-centred fundamental care. The RNs discussed their own responsibilities and leadership, particularly as regards who is responsible for what tasks on the ward.

The participants described that RNs must lead the delivery of nursing care. They noted that leadership was enabled when the responsibilities of the care team members, including the patients, were made clear. The RNs described themselves as having the roles of inviting patients to participate in meeting their own care needs, supporting the entire team in understanding and sharing patients’ care needs and plans, and leading them in following routines. ‘*My role is to help the patient understand the risks regarding pressure damage (…) My role is also to construct a plan that is feasible for both parties.*’ (RN, individual written reflection).

The RNs requested leadership that took responsibility for and supported the implementation of person-centred routines in the wards. RNs highlighted the need to discuss nursing leadership on the ward. The discussion focused particularly on the responsibility for nursing care concerning the high-pressure situation that had existed on the wards for a long time as a result of a shortage of RNs and closed beds. The participants noted that this situation had led to the loss of some routines, such as the team report, and hindered the delivery of person-centred fundamental care. RNs stated that nurse managers needed to act as leaders of nursing care in daily practice in order to enable the delivery of fundamental care, for instance by not filling staffing gaps resulting from sick leave by working on the wards themselves. As one RN said:

*It makes no sense that nurse managers are supposed to put out fires—that they*,* on any given day*,* have to both coordinate and run things at the same time. It’s not sustainable in the long run. Because this is how I often think things are like*,* that fires are put out by some boss who steps in to do something else.* (RN, focus group interview)

RNs stated that one part of a supportive culture, and the leaders’ responsibility, is to plan for the different nursing roles and use nursing competence on the wards to enable the delivery of person-centred fundamental care. The participants highlighted the need to improve the use of specialist nurses’ competence to support newly graduated RNs who had started their careers in this highly specialized surgical setting, caring for patients with complex care needs. This can be exemplified by the following quote:

*It should be a given that if one of the goals [on the ward] is that if I have the competence needed to coach and can be a source of security for the new RNs*,* then (I) should be allowed to shoulder that responsibility … As a specialist nurse*,* I could help and coach*,* but if I arrive [for my shift on the ward] and have [responsibility for] six patients on my own (it’s impossible).* (RN, focus group interview)

The participants described the need for clear, consistent, and visible nursing leadership in daily work at both the organizational level and the individual level, to enable the delivery of person-centred fundamental care. The participants indicated that the (core of) nursing needed to be embedded in nurse managers and all RNs. The following was stated by an RN:

*[What’s needed is] …a clear sense of leadership*,* really. A supportive leadership. From the nurse manager all the way down to the assistant nurse manager and the RN*,* who is the care team manager. You know*,* maybe make it very clear what kind of workplace this is expected to be and what our standards are [for treatment] from the top*,* then.* (RN, focus group interview)

### Enhancing comprehension of person-centred care routines to embed the approach in everyday thinking and actions

The participants highlighted the importance of a person-centred approach to care. However, some participants also expressed a need to increase their knowledge and ability to understand the difference between person-centred and ‘traditional’ care. Some participants perceived difficulties with person-centred routines where the patients are expected to be involved in the procedures; examples included the team report or the team rounds at the bedside. These participants stated that they believed it would take more time to perform a handover between shifts at the patient’s bedside—with the same information being repeated to some patients over the course of several days—than to perform a handover at the nurses’ station. The same participants indicated that they would be unlikely to adopt a different routine, thus hindering the delivery of person-centred fundamental care to patients in the wards. As one RN put it:

*No*,* but I feel it wouldn’t quite work here at the ward. The pace is so fast.* (RN, focus group interview)

Situations were also observed where RNs had a person-centred approach and involved a patient in the care plan, reflecting the importance of an informative and adaptable approach from the RNs. One example was when a patient planned the rate and dosage for tube feeding together with the RN. ‘*The patient seems satisfied with the situation.*’ (NA, individual written reflection).

A lack of knowledge and motivation to deliver person-centred care was noted as a hindrance. In a written reflection, an attitude of futility was described as hindering care delivery. This attitude was associated with missed fundamental care and care needs exceeding the available knowledge and time resources. ‘*Everything happens quickly*,* time is of the essence*,* and you have to adjust the interaction with the patient accordingly.*’ (RN, individual written reflection) The nursing staff also reflected upon their own leadership and how to lead themselves to acquire sufficient knowledge and motivation to provide patients with person-centred care.

The participants indicated that not all team members had the same perceptions of person-centred care. For example, one participant noted that conducting nursing rounds and setting goals with the patients was pointless, as their colleagues frequently failed to adhere to the care plan on subsequent days. Some participants stated that the team rounds were a good routine, enabling the delivery of person-centred fundamental care. However, the participants noted that when staff lacked knowledge about person-centred care, the focus was often more on the computer than on the patients, thus hindering the delivery of such care. They concluded that it was important to have the person-centred approach embedded in the thinking and not to see any routine as a series of tasks to be completed.

*It wouldn’t matter very much if you’re standing in the room [with the patient] instead of in the office if all you’re going to do is look at computer screens.* (RN, focus group interview)

## Discussion

This study describes what enables and hinders the delivery of person-centred fundamental care in a surgical department. The findings highlight the importance of a culture of daily mentorship to ensure adherence to person-centred routines. Additionally, the findings emphasize the need for distinct nursing leadership that provides support for both daily practice routines and high-pressure situations, while fostering bedside leadership among RNs. The study also identifies the need to enhance comprehension of person-centred care routines, to embed the approach in everyday thinking and actions on the ward.

Several person-centred routines, such as team rounds and bedside shift handovers, had been introduced on the wards over the years. However, their use been scarce or had gradually declined. This limited adoption may reflect the inherent challenges of achieving sustainable changes in clinical practice. The results indicate that several factors jeopardized the sustainability of person-centred routines on the wards, despite efforts in implementing them. These factors included individual preferences and values, staffing difficulties, and a lack of support and available nursing leadership in daily bedside work. These are examples of the kinds of contextual factors, outlined in the Fundamentals of Care framework [3], that can influence the care provided in the complex care environment on surgical wards. Although the aim of this qualitative study was not to compare the wards, the findings revealed that individual staff members could choose to perform a routine, such as involving the patient in the handover between shifts, or omit it from their daily work. Therefore, differences between the included wards may have existed due to contextual factors specific to each ward, such as the influence of individual staff members on the implementation of routines. Moore et al. [32] similarly found that professionals’ attitudes presented a barrier to implementing person-centred care, as they often reverted to ‘usual care’ routines or lacked the necessary interest, knowledge, and commitment to provide a person-centred approach to care. Our findings highlight the need for a culture of daily mentorship, where healthcare professionals can remind each other to maintain the person-centred routines that had been adopted. However, our study also indicates that developing a person-centred culture requires a deeper understanding of the underpinning values and ethics of person-centred care. This cannot be achieved through a few days of education—such as the workshop package on person-centred fundamental care in the current department—but requires continuous facilitation to embed person-centred care into everyday practice [43, 44]. The educational initiative aimed to influence the healthcare context, particularly the culture, by enhancing the nursing staff’s understanding of their roles and responsibilities in patient care, as well as deepening their comprehension of what person-centred care truly entails. It is essential that each ward engages in ongoing discussions about individual roles and responsibilities (i.e., an aspect of *the context of care* dimension in the framework) [3] and recognizes that the responsibility for delivering care according to routines and job descriptions needs to be shared by all nursing staff. Following the educational initiative, it can be assumed that a cultural shift in the understanding of person-centred care may contribute to broader changes within the care environment moving forward.

Participants in the focus groups expressed a need for distinct and responsive leadership from nurse managers in their daily work on the wards. The participants identified this as a prerequisite to enabling, implementing, and sustaining person-centred routines. Bedside leadership is a professional expectation on all RNs in Sweden [45]. This form of leadership encompasses responsibility for the delivery and evaluation of nursing care, including supervision of NAs within the team. The findings of the present study highlight the critical role of nurse managers in supporting and influencing daily work and the culture changes required to implement new routines. Their influence is particularly important in fostering a culture that promotes and sustains bedside leadership among RNs. Leadership has been identified as a central contextual factor in the Fundamentals of Care framework for fostering teams toward integrated fundamental care [46]. The use of the framework has also been recognized as an opportunity, at the organizational level, to reinforce nursing leadership and strengthen RNs’ leadership in their daily work [2]. However, the contextual factors influencing care delivery are complex and multifaceted [3, 46]. Research shows that it can be difficult for nurse managers to articulate the importance of fundamental care delivery in terms of clear intentions and actions [43]. Additionally, healthcare professionals report uncertainty regarding their roles and responsibilities in care delivery [47]. The role of nurse managers in our context (i.e., ward-based) is multifaceted, and their many duties and responsibilities, which involve not only oversight of nursing care but also staffing and budgeting, might hinder them from prioritizing bedside leadership. Nevertheless, our findings highlight the importance of nurse managers reflecting on how fundamental care is valued, organized, and delivered within a ward. Insights from other institutions, as reported in the literature [44, 48], offer valuable knowledge that can inform such reflections, along with the findings from the present study.

However, research has reported how organizations struggle to implement person-centred care [21, 22]. Contributing factors mentioned by the participants in this study include knowledge and distinguishing person-centred care from what they described as ‘traditional care’. Encouragingly, the interactive workshops and observations in their own clinical practice appeared to initiate meaningful reflections on this distinction. Participants also reported needing further support to consistently apply person-centred routines, as the existing organizational culture often hindered their ambitions. This need for support highlights the importance of adopting a whole-system approach to the implementation and sustainability of person-centredness in practice [49]. To achieve person-centred fundamental care on the surgical wards, person-centredness must be embedded across all levels of the organization and enacted through everyday interactions within interprofessional teams. One limitation of the educational workshops was the lack of involvement from the interprofessional teams. This was a deliberate decision during the planning phase, based on the rationale that strengthening the delivery of fundamental care within the three wards was a necessary first step. The next step will be for the nurse managers to use the evidence on what enables and hinders person-centred fundamental care delivery within their specific wards. This process should actively involve nursing staff, as the results showed strong engagement for driving change among them. Combined with managers who take responsibility for person-centred care, nursing staff could provide a good foundation for success. This step must also incorporate an interprofessional approach [50] and account for contextual factors at the ward level [31, 51]. For example, individual RNs and NAs can influence factors at the ward level, such as the values underpinning nursing and following the person-centred routines within each care team. Factors with an influence at the hospital level tend to be more challenging for nursing staff [51].

As previously discussed, the results also show the need to strengthen the professional role of RNs. The RNs discussed their responsibility and leadership, particularly as regards who is responsible for which tasks on the ward. This could be discussed in relation to the devaluation of fundamental care and reports of missed nursing care [10, 13, 24]. Embedding the Fundamentals of Care framework into the department as part of a practice model for nursing care, which has been successfully implemented in other hospital settings [44], can be one step toward revaluing nursing care. This step could lead toward the goal of achieving person-centred fundamental care in the studied department. In implementing the framework, it is paramount to consider, as also recommended by Kitson and colleagues [18], that it cannot only be embedded in practice routines but also needs to be integrated throughout the thinking, reflection, and assessment processes of staff and leaders [33]. The participants reported that aspects of the culture of the surgical wards, such as lack of time, stress, and varied understanding, were factors hindering the achievement of person-centred care, leading to fragmented nursing care. The occurrence of missed nursing care (including surgical care) is well-documented and often affects patients’ fundamental care needs, such as pain management, comforting and educating patients, oral hygiene, and administering medication [10, 11, 52, 53]. The present findings provide nurse managers and nursing staff with an understanding of the organizational culture within the studied department. Having such knowledge is essential before attempting to implement new routines or models of care [54].

Based on our results, there is an evident need to regularly include the rationale for the implemented routines on nursing forum agendas at the ward level and to focus on a long-term approach to the change in and sustainability of the routines chosen. To succeed with implementation, a strategic implementation process that addresses the identified areas essential for delivering person-centred fundamental care is crucial [50]. However, we know that implementing person-centred care requires cultural and organizational changes [22, 55], including considering factors linked to individuals and the workplace that should be changed. We also know that a shared culture and structural understanding, including a long-term approach, are important aspects of a change management process [56]. The International Learning Collaborative Organization statement [57] highlights the need for healthcare systems to evolve so that all healthcare providers and leaders understand and value person-centred fundamental care and implement it to a consistently high standard.

### Strengths and limitations

One strength of the study is the data collection, which included many from the bedside nursing staff and used multiple methods to gain understanding of the complex phenomenon being analysed. Sentences from flip charts, which emerged from small group discussions following short lectures, individual written reflections from observations during clinical practice, and recorded interviews with discussions in groups, captured many different experiences among the nursing staff. The focus group interviews included a well-balanced distribution of participants in terms of both years in the profession and years at their workplace, which suggests that we were likely to identify a wide range of enablers and hindrances to the delivery of person-centred fundamental care. The preunderstanding of the context among the authors performing the data collection and analysis facilitated that work. In addition, some of the authors who participated in discussing the results did not have experience in surgical care, which provided further perspectives on the data, thereby adding to the trustworthiness of the study. Discussing the results with all the authors (to reach consensus) and using illustrative quotes ensured that the results were directly derived from the collected data. A limitation of the study is the setting: a single department in a university hospital. However, this was a large department, and a thorough description of the context can allow the reader to assess the transferability of findings to similar settings.

The numbers of flip charts and written reflections collected and focus group interviews performed were not based on data saturation but rather on the number of nursing staff attending the workshops and interviews during performance of the study. In content analysis, saturation is not the goal. However, there must be sufficient data to account for significant variations. The optimal amount of data depends on the data quality and the aim of the study [2]. As our results highlight various challenges and possibilities of implementing person-centred care, as well as corresponding with some previous research. Therefore, we believe that the amount of data may be considered sufficient.

The Fundamentals of Care framework was used as the theoretical foundation in the study. The framework is feasible for use in clinical settings [2, 44, 48], and is recognized by RNs across various countries as both easy to understand and clinically relevant [2]. Consequently, it was considered to support the learning of person-centred fundamental care among the nursing staff participating in the workshop activities. No assessment of participants’ understanding of the framework was conducted. This was not part of the rationale for the present study, but limits the possibilities for interpretation of the workshops’ educational impact. However, the narratives written by the participants in their individual reflections on person-centred fundamental care delivery in situations in clinical practice indicated that they had, to some extent, embedded the framework into their thinking. Additionally, data from the group discussion summaries showed that participants utilized the framework when proposing developments of person-centred routines on the wards.

## Conclusion

To provide person-centred fundamental care, nursing staff need organizational support in both culture and routines. Nurse managers need to be clear on how care is to be delivered, challenge prevailing old habits, and act as role models to change practice. Nursing staff need to take their responsibility by adhering to routines and contributing to driving changes. The results can be used to navigate the complex implementation of person-centred fundamental care and also demonstrate the need for a change toward person-centred fundamental care being valued and prioritized in organizations.

## Supplementary Information

Below is the link to the electronic supplementary material.

Supplementary Material 1

## Data Availability

Access to data is restricted. If people familiar with the context in which the study is carried out were to read the entire interviews, they would recognize the interviewees. Therefore, due to the rules of the GDPR and the Swedish Ethical Review Authority, we cannot make our data available.
