# Supplementary material for: What enables and hinders nursing staff in delivering person-centred fundamental care? A qualitative study within the incharge programme
Source: BMC Nurs. 2025 Oct 31;24:1361. doi: 10.1186/s12912-025-04038-0 (PMC12577148; doi:10.1186/s12912-025-04038-0)
Supplement: Supplementary file 1 — Supplementary Material 1 [file 12912_2025_4038_MOESM1_ESM.docx]

# Questions, and interview guide

## Step I, Questions during interactive workshops, summarised on flip charts

1. What person-centred care routines exist on the ward?
2. What enablers and hindrances are related to the identified routines?
3. What is your role in delivering person-centred fundamental care?
4. What person-centred care routines do you want to continue working with at your ward?

5. What is your role in achieving these person-centred care routines?

## Step II, Questions for the individual written reflections on a situation in the participant's clinical practice

1. Describe the situation.
2. What is person-centred in the situation?
3. What is not person-centred in the situation?
4. What enablers are there to make the situation person-centred?
5. What hindrances are there to make the situation person-centred?
6. What is my role as RN/NA to make the situation person-centred?

## Step III, Questions during interactive workshops, summarised on flip charts

1. What do you want the ward to develop further?

3. What is required to succeed?

## Step IV, Interview guide focus group interviews

The guide focuses on what enables and hinders nurses’ delivery of person-centred fundamental care in four areas. Including different questions within each question area, for each ward, depending on the results from collected data in steps I & III. The aim of the interview was placed on the table; Identify how the ward can further develop person-centered care to enhance fundamental nursing care.

**Presenting results from step I & III**

During workshops 1 and 2, RNs and NAs from your ward provided suggestions, on what you would like to continue working on regarding person-centered routines and approaches.

**Question area 1: Person-centred approach**

What do you think about the following aspects, regarding person-centred approach, that was raised during the workshops?

Ward A

- Non-hierarchical and well-functioning team.
- Routines and communication within the team including the patient as a partner.
- The importance of empathy in patient interaction.

Ward B

- Understand the difference a person-centred approach makes for the patient.
- Invite the patient as a partner, including communication and information.

Ward C

- Invite the patient to tell their story and be involved in their care.
- Utilize patients' experience data, from the ward, to evaluate and improve nursing care.

**Question area 2: Person-centred routines**

What do you think about the following aspects regarding person-centred routines, that were raised during the workshops?

Ward A.

- Patient whiteboard, team round.
- Information to patients.
- Support of function staff.

Ward B

- Bedside handover, team round, nursing round, patient diary, and patient whiteboard.
- Nursing care plans (during the hospital stay and discharge), including information, documentation, and risk assessment.

Ward C

- Team round, team board, bedside handover, hourly rounding.
- Assessment and registration of vital signs.
- Information to the patient, and coordination at discharge.

**Question area 3: Prerequisites to taking a person-centred approach at work**

What do you think about the following aspects, regarding prerequisites to work person-centred, that were raised during the workshops?

Ward A

- Continuous education, time for reflection, continuity of care, resources, and available equipment.
- Patient information in different languages.

Ward B

- Communication within the team, a culture with an open climate.
- Education and introductions to new employees.
- Good working environment and work conditions.

Ward C

- Communication within the team, a culture with an open climate.
- Time for reflection, education.
- Lower nurse-patient ratio.

**Question area 4: Roles in the ward to strengthen fundamental care**

What do you think about the following aspects, regarding roles at the ward to strengthen fundamental care, that were raised during the workshops?

Ward A

- Create a non-hierarchical well-functioning team with explicit roles and responsibilities.
- Utilize competencies.

Ward B

- Utilize competencies.
- Distinct leadership.

Ward C

- Utilize competencies of RN and NA.

**Each area was also discussed in terms of relevance and feasibility for each ward**

The areas that were raised and discussed during this interview are:

- relevant for your ward?
- feasible at your ward?

**Closing up the interview**

The interviewer summarizes what the interview has been about and what has been said.

- Do you agree with the summary?
- Is there anything you would like to add?

## Researchers' competence in workshop facilitation and data analysis

AKG - Senior lecturer, with teaching comprising a substantial part of the position. PhD in nursing at the university, female researcher with experience in qualitative research methods.

TA – Nursing leader, Department of Surgery, with extensive experience in teaching. PhD in nursing at the present surgical department, a female researcher with experience in qualitative research methods.

EJ - Senior lecturer, with teaching comprising a substantial part of the position. Associate professor in nursing at the university with a joint position at the present surgical department, female, researcher with extensive experience in qualitative research methods.

AH, LN, KE, EF, EB – All PhD with experience in qualitative research methods.
